# Human herpesvirus 8 replicates in primary B lymphocytes and induces polyfunctional cytokine and chemokine responses

**DOI:** 10.1186/1750-9378-7-S1-P36

**Published:** 2012-04-19

**Authors:** Emilee Knowlton, Giovanna Rappocciolo, Frank J Jenkins, Mariel Jais, Paolo Piazza, Charles R Rinaldo

**Affiliations:** 1 Department of Infectious Diseases and Microbiology, Graduate School of Public Health, University of Pittsburgh, Pittsburgh, PA, USA

## Objectives

We have previously shown that DC-SIGN expressing, activated B cells support human herpesvirus 8 (HHV-8; KSHV) replication. Cytokines and chemokines play an important role in KS, including tumor-cell proliferation, angiogenesis and vascular permeability. We therefore examined virus replication in relation to production of soluble immune mediators by HHV-8 infected B cells.

## Methods

B cells were loaded with purified “live” HHV-8, purified UV-light inactivated HHV-8 (UV-HHV-8) or soluble HHV-8 glycoprotein B (gB). HHV-8 replication was measured by real time PCR for viral DNA and intracellular staining (ICS) and flow cytometry for the viral lytic proteins ORF59 and K8.1. ICS was used to assess cell-associated, polyfunctional cytokine-chemokine production, and B cell supernatants were tested for cytokine/chemokine secretion by Cytometric Bead Array (BD Biosciences).

## Results

ORF59 and K8.1 HHV-8 lytic proteins were detected in subpopulations of B cells expressing IgM, CD19, CD20, CD23 and CD38 surface antigens. The percentage of ORF59 and K8.1 positive B cells and level of viral DNA increased over several days of infection. The lytically infected B cells were more polyfunctional and produced more cell-associated TNF-α, IL-6, IL-8, MIP-1α and MIP-1β than the uninfected cultures. Significant increases in the 5 immune mediators were also detected in the supernatant of HHV-8 treated cultures by 24 hours, which were 16-100 fold higher than in untreated B cell cultures. Treatment of the B cells with UV-HHV-8 or gB induced a similar pattern of cytokine and chemokine production in the supernatant as did live HHV-8.

## Conclusions

This is the first evidence of HHV-8 replication in primary B cells by both flow cytometry and viral DNA analysis, together with polyfunctional B cells producing multiple immune mediators, over the course of in vitro HHV-8 infection. There was similar production of cytokines and chemokines induced by live HHV-8, UV-HHV-8 and HHV-8 gB, indicating virus binding via gB alone is sufficient to elicit a B cell immune response to HHV-8. We are currently delineating B cell subsets that support HHV-8 lytic infection and investigating the role of individual cytokines and chemokines on HHV-8 replication.

